# Ambient Ozone Exposure and Pneumothorax Risk After CT-Guided Lung Biopsy

**DOI:** 10.3390/tomography12070098

**Published:** 2026-07-01

**Authors:** Nour Afilal, Alois Komarek, Michael Dieckmeyer, Elif Can, Martin Jonczyk, Johannes T. Heverhagen, Michael P. Brönnimann

**Affiliations:** 1 Department of Diagnostic, Interventional and Pediatric Radiology, Inselspital, Bern University Hospital, University of Bern, Rosenbühlgasse 27, 3010 Bern, Switzerland; nour.afilal@insel.ch (N.A.); alois.komarek@insel.ch (A.K.); michael.dieckmeyer@zgks.ch (M.D.); johannes.heverhagen@insel.ch (J.T.H.); 2 Service of Radiology, Geneva University Hospital, 1211 Geneva, Switzerland; 3 Department of Interventional Radiology, Medical Center–University of Freiburg, Faculty of Medicine, University of Freiburg, 79110 Freiburg im Breisgau, Germany; elif.can@uniklinik-freiburg.de; 4 Department of Radiology, Charité–Universitätsmedizin, Augustenburger Platz 1, 13353 Berlin, Germany; martin.jonczyk@charite.de; 5 Division of Interventional Radiology, Department of Radiology, Stanford University School of Medicine, Stanford, CA 94305, USA

**Keywords:** pneumothorax, biopsy, computed tomography, air pollutants, environmental exposure, ozone

## Abstract

Background/Objectives: To evaluate whether day-of-procedure ambient ozone exposure is associated with pneumothorax after CT-guided lung biopsy. Methods: This retrospective single-centre study included 160 CT-guided lung biopsies performed between January 2018 and February 2026. Environmental data from the day of biopsy were assigned from the nearest national monitoring station. The primary outcome was any pneumothorax on post-biopsy CT; the secondary outcome was drainage-requiring pneumothorax. Multivariable logistic regression included ozone exposure, emphysema, and access route through dependent lung area (ARDA). Ozone was analysed as a continuous variable per 10 μg/m^3^ and, exploratorily, using a ROC-derived threshold of ≥75.8 μg/m^3^. Restricted cubic splines assessed nonlinearity. Sensitivity models adjusted for needle size, biopsy system, operator identity, and season. Drainage-requiring pneumothorax was analysed using Firth logistic regression. Results: Pneumothorax occurred after 86 of 160 biopsies (53.8%), and 13 biopsies (8.1%) required drainage. Ozone was not associated with pneumothorax when modelled linearly (OR, 1.09 per 10 μg/m^3^; 95% CI, 0.97–1.23; *p* = 0.167). In exploratory threshold modelling, ozone ≥ 75.8 μg/m^3^ was associated with pneumothorax (OR, 2.76; 95% CI, 1.39–5.61; *p* = 0.004). Emphysema increased pneumothorax odds (OR, 2.16; 95% CI, 1.03–4.68; *p* = 0.047), whereas ARDA was protective (OR, 0.23; 95% CI, 0.11–0.45; *p* < 0.001). Spline analysis supported nonlinearity (*p* = 0.001). For drainage-requiring pneumothorax, only emphysema was significant. Conclusions: Ambient ozone showed an exploratory nonlinear association with pneumothorax after CT-guided lung biopsy, with a threshold signal around 70–80 μg/m^3^. ARDA was protective, whereas emphysema was associated with drainage-requiring pneumothorax.

## Simple Summary

Pneumothorax is a common complication after computed tomography-guided lung biopsy, but most known risk factors relate to the patient’s lung structure or the biopsy technique. This study examined whether ambient ozone levels on the day of biopsy were associated with pneumothorax risk. Higher ozone exposure showed an exploratory nonlinear association with post-biopsy pneumothorax, while access through dependent lung areas appeared protective, and emphysema was linked to more clinically relevant cases requiring drainage. These findings suggest that environmental exposure may influence biopsy risk and should be investigated further in larger multicentre studies.

## 1. Introduction

CT-guided lung biopsy (CTLB) is an established technique for evaluating thoracic lesions, with reported diagnostic accuracy ranging from 83% to 97% [1]. As targeted therapies and immunotherapies expand the clinical need for histological and molecular tissue characterisation, the number of image-guided lung biopsies continues to increase [2,3]. Pneumothorax remains the most frequent complication, with reported rates varying widely depending on study design, patient characteristics, and procedural technique [4,5,6,7]. Established risk factors are mainly technical or anatomical, including lesion size, lesion depth, needle size, pleural angle, fissure traversal, and patient positioning [8,9,10,11,12,13,14,15]. Among patient-related factors, age and emphysema have been reported most consistently [8,16,17]. These factors are clinically relevant because pneumothorax may result not only from external air entry through the needle tract, but also from alveolar rupture and propagation of air leakage, highlighting the role of structural lung vulnerability [7]. Air pollutants may increase lung vulnerability through inflammatory and functional effects. Short-term exposure to ozone and nitrogen oxides has been associated with respiratory symptoms, airway hyperresponsiveness, epithelial injury, and alveolar inflammation [2,18,19,20,21]. Associations between air pollutants and spontaneous pneumothorax have been reported, although findings remain inconsistent [22,23,24,25]. Meteorological factors, including atmospheric pressure, temperature, and humidity, have also been investigated in relation to primary spontaneous pneumothorax, but the evidence is mixed [2,23,24,25]. In contrast, environmental determinants of pneumothorax after CTLB remain largely unexplored. Only one previous study evaluated meteorological conditions in the CTLB setting and found no association with pneumothorax [12]. Given these uncertainties, we hypothesised that short-term environmental exposure, particularly ambient ozone on the day of biopsy, may be associated with pneumothorax after CTLB. The aim of this study was to evaluate the association between day-of-procedure environmental exposure and pneumothorax risk after CTLB, while accounting for established patient-related and procedural factors, including emphysema and access through dependent lung areas. We additionally assessed whether any observed ozone association was linear, threshold-dependent, and robust to adjustment for technical, operator-related, and seasonal factors.

## 2. Materials and Methods

### 2.1. Study Population

This retrospective single-centre study included consecutive adult patients who underwent CT-guided lung biopsy at a Swiss university hospital between January 2018 and February 2026. Biopsies were performed for indeterminate or suspicious pulmonary lesions when tissue sampling was clinically indicated and transbronchial biopsy was considered unsuitable or had been non-diagnostic. The study was approved by the local ethics committee and was conducted in accordance with institutional requirements.

**Figure 1:**
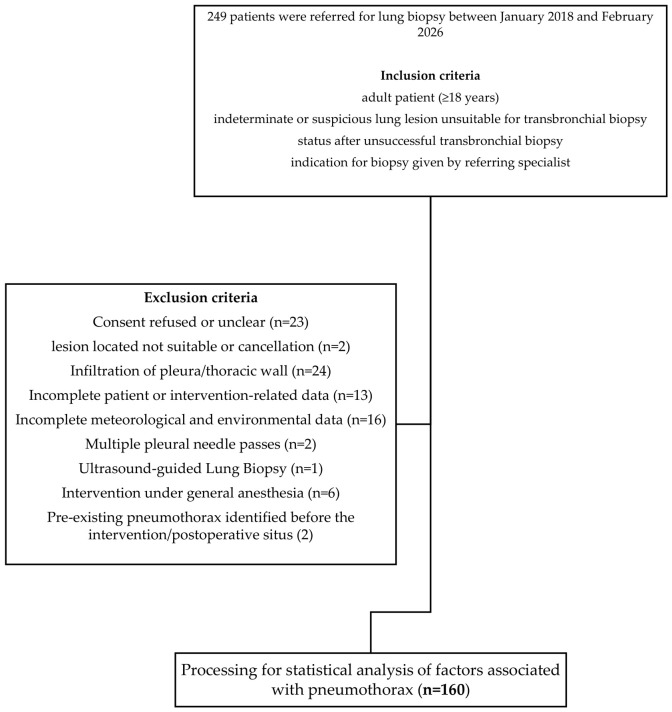
Study cohort selection for pneumothorax risk analysis following lung biopsy.

Exclusion criteria were defined to reduce confounding from altered pleural mechanics or substantially different procedural conditions. Cases were excluded if lesions showed pleural or chest wall infiltration, if multiple pleural needle passes were performed, if postoperative lung anatomy or preexisting pneumothorax was present, if the procedure was performed under general anaesthesia, if relevant clinical, procedural, meteorological, or environmental exposure data were missing, or if the biopsy was performed under ultrasound rather than CT guidance. The final analysis cohort comprised 160 CT-guided lung biopsies (Figure 1).

### 2.2. Baseline Evaluation and Biopsy Technique

All patients underwent routine clinical assessment and laboratory testing before biopsy. Coagulation requirements included an international normalised ratio < 1.5 or Quick value > 60%, haemoglobin > 80 g/L, and platelet count > 50 × 10^9^/L. Antithrombotic medication was paused according to institutional guidelines.

Biopsies were performed by experienced interventional radiologists using a coaxial technique. Procedures were performed on CT systems, including an Asteion 4SL scanner (Toshiba Medical Systems, Otawara, Japan) and a SOMATOM X.cite scanner (Siemens Healthineers, Forchheim, Germany), using thin-section non-contrast planning images. Depending on lesion location and operator preference, 15-, 17-, or 19-gauge coaxial introducers were used with 16-, 18-, or 20-gauge biopsy devices (SemiCut side-cutting devies; Medical Devices Lease S.A., Zug, Switzerland or CorVocet^TM^ full core devices; Merit Medical Systems, South Jordan, UT, USA). Biopsy trajectories were planned to avoid fissures and visible vessels whenever possible. Patient position was selected by the operator according to lesion location, expected access route, and procedural safety.

Local anaesthesia was administered with 1% lidocaine (Lidocain Streuli, Streuli Pharma AG, Uznach, Switzerland). Routine sedation and standardised breath-hold commands were not used. After tissue acquisition, the needle was removed without tract sealing. Immediate and 5-mine post-biopsy CT controls were obtained. Patients were monitored after biopsy according to institutional practice. Progressive pneumothorax prompted placement of a 6- or 8-F pleural drainage catheter (Flexima drainage catheter, Boston Scientific Corporation, Marlborough, MA, USA). Stable pneumothorax was followed clinically and by chest radiography when indicated.

The primary outcome was any pneumothorax, defined as intrapleural gas detected on post-biopsy CT after needle removal. The secondary clinically relevant endpoint was drainage-requiring pneumothorax, defined as pneumothorax requiring placement of a pleural drainage catheter after CT-guided biopsy.

### 2.3. Imaging Review and Procedural Variables

All procedures were reviewed in the institutional picture archiving and communication system (PACS; Sectra IDS7, version 27.1, patch 19/2025; Sectra AB, Linköping, Sweden) by radiologists who were not involved in the interventions. Recorded variables included age, sex, patient position, lesion size, lesion location, pleura-to-lesion distance, skin-to-lesion distance, biopsy angle, needle size, biopsy system, number of samples, respiratory comorbidity, emphysema, and histopathological diagnosis.

**Figure 2:**
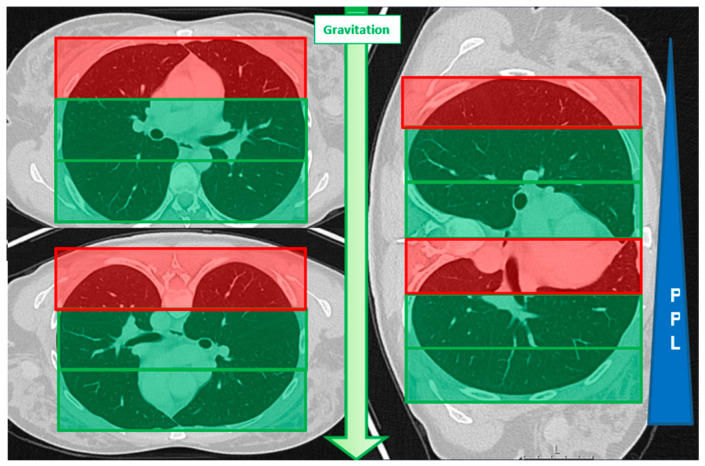
Schematic illustration of the zoning used for this study according to position-dependent gravitational effect on pleural pressure (PPL). For zoning, we applied the rule of thirds. Only the zone “RED” was determined as non-dependent. The green zones represent the dependent lung regions.

A simplified gravitational zoning model was applied to classify whether the access route began in a dependent lung region (ARDA) (Figure 2). To assess reproducibility, ARDA classification was independently repeated by a second radiologist blinded to pneumothorax outcome and environmental exposure. Inter-observer agreement for the binary ARDA classification was assessed using Cohen’s kappa. Discrepant cases were resolved by consensus, and the consensus classification was used for the final analysis. Emphysema was visually assessed as present or absent. A binary emphysema variable was used to avoid unstable subgroup estimates from small emphysema severity strata.

### 2.4. Meteorological and Air Pollution Data

Meteorological and air pollution exposure data were assigned according to the intervention site in Bern because individual patient-level exposure before biopsy could not be reliably reconstructed. Individual data on residence-specific exposure, commuting route or mode, time spent indoors before biopsy, ventilation conditions, and indoor ozone attenuation were not available in the retrospective medical records. Meteorological variables included temperature, dew point, humidity, wind speed, atmospheric pressure, and precipitation. Season and intervention timing were recorded to account for seasonal and diurnal variation. Urban residence was defined according to municipality size based on the medical record.

Air pollution data were obtained from the nearest monitoring station of the National Air Pollution Monitoring Network, located at Bern Bollwerk, for the day of intervention. Ozone was recorded as the maximum hourly mean concentration on the day of biopsy. Other pollutants, including NO_2_, CO, PM_10_, PM_2.5_, elemental carbon, condensation particle concentration, and NO_x_, were recorded as daily averages. Ozone was selected as the primary environmental exposure of interest based on biological plausibility and prior literature linking ozone exposure to respiratory vulnerability and spontaneous pneumothorax.

### 2.5. Statistical Analysis

Analyses were performed in R, version 4.5.3 (R Foundation for Statistical Computing, Vienna, Austria). Continuous variables were summarised as median [interquartile range] and categorical variables as *n*/*N* (%). Group comparisons between biopsies with and without pneumothorax were performed using Wilcoxon rank-sum tests for continuous variables and chi-square tests for categorical variables. Two-sided *p*-values < 0.05 were considered statistically significant.

The primary multivariable model was conceptually defined and included three clinically motivated predictors: ozone exposure, emphysema, and ARDA. Ozone was analysed first as a continuous linear variable scaled per 10 μg/m^3^, using the maximum hourly mean concentration on the day of biopsy. Because no externally validated ozone threshold exists for post-biopsy pneumothorax, an exploratory threshold analysis was performed using a ROC-derived cutoff of ≥75.8 μg/m^3^ for the maximum hourly mean ozone concentration. This cutoff was considered hypothesis-generating and was further evaluated by threshold sensitivity analyses across alternative cutoffs. A Firth penalised logistic regression model was additionally fitted for the threshold model as a robustness analysis.

Because the linear ozone term and the threshold model suggested a possible nonlinear exposure-response relationship, restricted cubic spline modelling was performed. The spline model was adjusted for emphysema and ARDA and restricted to the 5th to 95th percentile of ozone exposure to reduce the influence of sparse extreme values. Threshold sensitivity analyses were performed using alternative ozone cutoffs from 60 to 100 μg/m^3^ for the main figure and from 60 to 120 μg/m^3^ for the supplementary analysis. Each threshold model included ozone, emphysema, and ARDA.

Robustness analyses assessed whether the ozone association persisted after adjustment for procedural or contextual covariates. Separate sensitivity models adjusted the base threshold model for needle size, biopsy system, operator identity, and season. Calendar year was not included in the main robustness table because same-day ozone exposure was directly measured and season was considered the more biologically relevant temporal factor. To further address potential confounding by procedural technique and operator-related practice patterns, an extended sensitivity model included ozone ≥ 75.8 μg/m^3^, emphysema, ARDA, needle size, biopsy system, operator identity, and season simultaneously. A second extended model additionally included lesion size and pleura-to-lesion distance. Because the number of operator categories was relatively high, a sparse operator model was also fitted in which individual operator identity was replaced by a binary indicator of above-median operator-specific pneumothorax rate. These extended models were reported as supplementary analyses. Drainage-requiring pneumothorax was analysed using Firth penalised logistic regression because of the limited number of drainage events.

## 3. Results

### 3.1. Study Cohort and Baseline Characteristics

A total of 160 CT-guided lung biopsies met the inclusion criteria and were included in the final analysis (Figure 1). Pneumothorax occurred in 86 of 160 biopsies, corresponding to an overall pneumothorax rate of 53.8%. Drainage-requiring pneumothorax occurred in 13 of 160 biopsies, corresponding to 8.1%. No patient required surgical intervention. Baseline, lesion-related, procedural, and selected environmental characteristics stratified by pneumothorax occurrence are summarised in Table 1. Patient age, sex, patient position, lesion size, lesion location, pleura-to-lesion distance, skin-to-lesion distance, biopsy angle, number of samples, season, urban residence, and intervention after noon showed no statistically significant differences between biopsies with and without pneumothorax. Malignant lesions represented the majority of biopsied targets.

ARDA was more frequent among biopsies without pneumothorax. Inter-observer agreement for the binary ARDA classification was substantial, with a Cohen’s kappa of κ = 0.80. Emphysema was more common in biopsies complicated by pneumothorax. Ozone concentrations were higher among biopsies with pneumothorax, and procedures performed at ozone levels ≥ 75.8 μg/m^3^ were more frequent in the pneumothorax group. Technical factors, including needle size and biopsy system, showed group-level differences and were therefore included in robustness analyses. Other measured air pollutants and meteorological variables did not show robust associations with pneumothorax after adjustment and were not included in the primary models.

### 3.2. Primary Multivariable Models

In the primary model treating ozone as a continuous linear variable, ozone per 10 μg/m^3^ was not significantly associated with pneumothorax, although the effect direction was positive (OR 1.09; 95% CI, 0.97–1.23; *p* = 0.167). Emphysema showed a positive but borderline association, whereas ARDA was significantly associated with lower pneumothorax odds (Table 2).

In the exploratory threshold model using the ROC-derived cutoff of ≥75.8 μg/m^3^, elevated ozone was associated with pneumothorax. The adjusted OR for ozone ≥ 75.8 μg/m^3^ was 2.76 (95% CI, 1.39–5.61; *p* = 0.004). Emphysema was also associated with increased pneumothorax odds (OR 2.16; 95% CI, 1.03–4.68; *p* = 0.047). ARDA was associated with lower pneumothorax odds (OR 0.23; 95% CI, 0.11–0.45; *p* < 0.001). Firth penalised regression yielded comparable estimates (Table 2).

### 3.3. Nonlinear Ozone Association and Threshold Sensitivity

**Figure 3:**
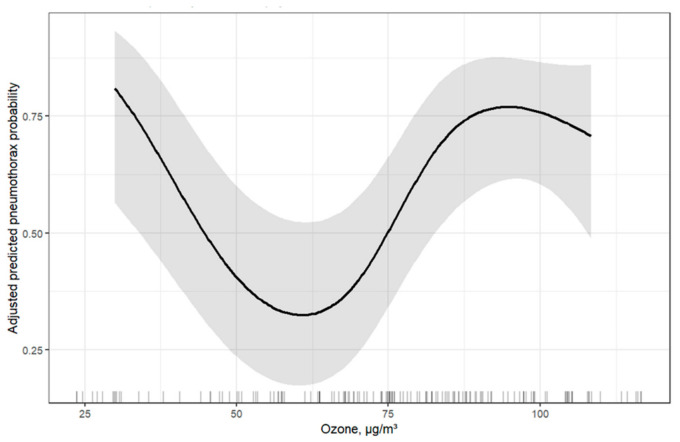
Adjusted nonlinear association between ambient ozone and pneumothorax risk. Restricted cubic spline showing the adjusted predicted probability of pneumothorax according to maximum hourly mean ambient ozone concentration on the day of CT-guided lung biopsy. The analysis was restricted to the 5th to 95th percentile of ozone exposure to reduce the influence of sparse extreme values. The model was adjusted for emphysema and access route through dependent lung area (ARDA). Shaded areas represent 95% confidence intervals. ARDA = access route begins in dependent area according to our model; CT = computed tomography.

Given the absence of a significant linear ozone effect and the positive threshold model, restricted cubic spline analysis was performed to assess nonlinearity. The adjusted spline model demonstrated a significant nonlinear association between ozone and pneumothorax risk. After restriction to the 5th to 95th percentile of ozone exposure, the overall ozone term was significant (*p* = 0.002), and the nonlinear component was also significant (*p* = 0.001). The adjusted probability curve showed increasing pneumothorax probability above approximately 70–80 μg/m^3^, while estimates at exposure extremes were less precise (Figure 3).

**Figure 4:**
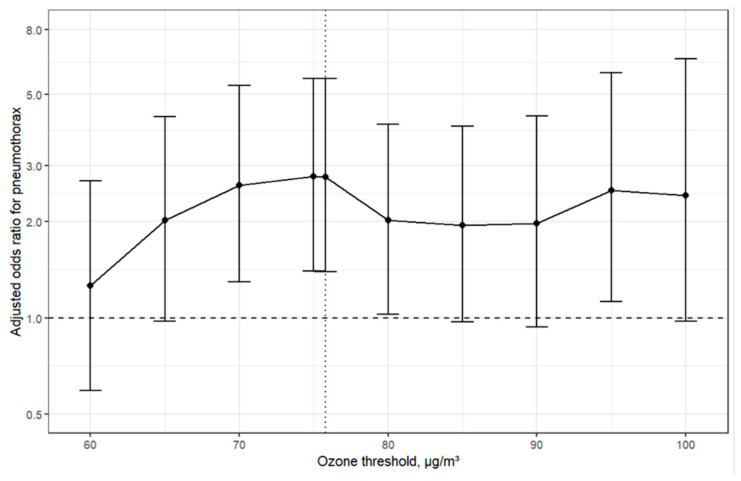
Threshold sensitivity analysis for ambient ozone exposure. Adjusted odds ratios for pneumothorax across alternative cutoffs of maximum hourly mean ambient ozone concentration ranging from 60 to 100 μg/m^3^. Each logistic regression model included the respective ozone threshold, emphysema, and access route that begins in the dependent area according to our model (ARDA). Error bars represent 95% confidence intervals. The dashed horizontal line indicates an odds ratio of 1. The vertical dotted line marks the ROC-derived ozone threshold of 75.8 μg/m^3^. CI = confidence interval; OR = odds ratio; ROC = receiver operating characteristic.

Threshold sensitivity analysis was performed using alternative ozone cutoffs from 60 to 100 μg/m^3^. Across these cutoffs, adjusted odds ratios were consistently above 1. The most precise estimates were observed around 70–80 μg/m^3^, including the exploratory ROC-derived threshold of 75.8 μg/m^3^. This supported a threshold signal in this exposure range rather than a uniquely defined biological cutoff (Figure 4). In the full supplementary threshold analysis, estimates at very high cutoffs remained directionally positive but were imprecise because of small numbers of procedures above these exposure levels (Table S1).

### 3.4. Robustness Analyses

The association between ozone ≥ 75.8 μg/m^3^ and pneumothorax remained stable across sensitivity models adjusting for selected procedural and contextual variables (Table 3). In the base threshold model, ozone ≥ 75.8 μg/m^3^ was associated with pneumothorax with an OR of 2.76. After additional adjustment for needle size, the effect estimate was 2.87. After adjustment for biopsy system, the effect estimate was 2.93. The ozone association also remained present after adjustment for operator identity (OR 2.53) and after adjustment for season (OR 2.96).

ARDA remained associated with lower pneumothorax odds across all robustness models, with adjusted odds ratios ranging from approximately 0.22 to 0.27 (Table 3). Emphysema remained directionally associated with higher pneumothorax odds across all models, although statistical significance varied after additional adjustment. Operator distribution and pneumothorax rates are shown in Table S2.

In an extended sensitivity model including needle size, biopsy system, operator identity, and season simultaneously, ozone ≥ 75.8 μg/m^3^ remained associated with pneumothorax (OR 2.93; 95% CI, 1.12–7.97; *p* = 0.030). Similar estimates were observed after additional adjustment for lesion size and pleura-to-lesion distance (OR 2.91; 95% CI, 1.11–7.92; *p* = 0.032) and in a sparse operator model (OR 2.57; 95% CI, 1.05–6.49; *p* = 0.039) (Table S3).

### 3.5. Drainage-Requiring Pneumothorax

Drainage-requiring pneumothorax was analysed as a clinically relevant secondary endpoint. Because 13 drainage events occurred, Firth penalised logistic regression was used. In this model, emphysema was associated with drainage-requiring pneumothorax (OR 3.62; 95% CI, 1.19–11.87; *p* = 0.023). Ozone ≥ 75.8 μg/m^3^ was not significantly associated with drainage requirement (OR 1.63; 95% CI, 0.53–5.41; *p* = 0.399), and ARDA was not significantly associated with this endpoint (OR 0.85; 95% CI, 0.26–2.63; *p* = 0.778) (Table 4).

## 4. Discussion

This study evaluated whether short-term environmental exposure is associated with pneumothorax after CT-guided lung biopsy. Using a conceptually defined model, ozone exposure showed an exploratory nonlinear association with any pneumothorax, with a threshold signal around 70–80 μg/m^3^. Emphysema was associated with both any pneumothorax and drainage-requiring pneumothorax, whereas ARDA was the most consistent protective procedural factor for any pneumothorax. The main contribution of this study is the identification of a previously unrecognised short-term environmental exposure signal in the setting of CT-guided lung biopsy. Although exploratory and not clinically actionable, this ozone association suggests that procedural pneumothorax risk may be influenced not only by static anatomical and technical factors, but also by transient environmental conditions that may affect lung parenchymal vulnerability. The restricted modelling strategy was chosen to preserve this hypothesis-generating focus while reducing overfitting and avoiding extensive data-driven variable selection in a moderate-sized cohort.

Ozone showed a consistent association with pneumothorax in threshold-based analyses. Although ozone was not significant when modelled as a linear continuous exposure, restricted cubic spline analysis demonstrated a significant nonlinear association, and threshold models showed stable positive associations across clinically plausible cutoffs. This suggests that, in this cohort, the relationship between ambient ozone and post-biopsy pneumothorax was better captured by nonlinear and exploratory threshold modelling than by a simple linear term. Prior research has demonstrated that short-term air pollution exposure can trigger acute respiratory reactions, including airway hyperresponsiveness and inflammation [18,26]. Elevated ozone levels have also been linked to spontaneous pneumothorax in several clinical settings [22]. Experimental and clinical studies suggest that ozone exposure may induce oxidative stress, epithelial injury, and airway inflammation [27,28]. In the context of CT-guided lung biopsy, it is biologically plausible, but unproven, that such effects could transiently increase lung vulnerability through mechanisms such as airway narrowing, air trapping, or alveolar overdistension. However, the present retrospective study did not include biomarkers of oxidative stress or inflammation, lung function testing, or direct measures of alveolar mechanics. Therefore, this proposed pathway remains hypothetical and requires validation in dedicated mechanistic studies. A possible conceptual pathway is shown in Figure S1.

The observed association between emphysema and pneumothorax aligns with previous reports describing structural vulnerability of emphysematous lung tissue during biopsy [16,17]. Emphysematous regions often display decreased elasticity and increased air retention, which may increase the likelihood of air leakage after pleural and parenchymal puncture. In the present study, emphysema was associated not only with any pneumothorax in the primary threshold model but also with drainage-requiring pneumothorax in Firth penalised regression. This finding is consistent with emphysema acting as an intrinsic marker of lung vulnerability, particularly for clinically relevant pneumothorax requiring drainage. ARDA demonstrated a strong protective association with any pneumothorax, consistent with physiological principles related to gravitational compression and reduced alveolar overdistension in dependent lung zones [14,29]. Several studies have reported that dependent positioning or dependent access routes reduce pneumothorax risk during CTLB [13,14,29,30]. Experimental data further suggest that dependent positioning lowers pressure gradients between alveoli and pleura, which may limit the propagation of small parenchymal defects [31]. In the present analysis, ARDA remained protective across sensitivity models adjusting for needle size, biopsy system, operator identity, and season. Illustrative biopsy cases are shown in Figure S2. The overall pneumothorax rate in this cohort was higher than in some previous CT-guided lung biopsy series, likely reflecting the sensitive endpoint definition.

The ozone threshold identified in this study, 75.8 μg/m^3^ as maximum hourly mean concentration on the day of intervention, falls below the Swiss legal short-term limit of 120 μg/m^3^ and corresponds to moderate short-term exposure according to the Swiss short-term air pollution index [32,33]. However, this threshold was derived from the present cohort and should be interpreted as hypothesis-generating rather than as a regulatory or clinical action threshold. The threshold sensitivity analysis showed directionally consistent positive associations across several cutoffs, particularly from approximately 70 to 100 μg/m^3^, whereas estimates at very high cutoffs were imprecise because of sparse exposure strata. Multicentre cohorts with broader exposure gradients are needed to determine whether ozone thresholds can contribute to procedural risk stratification. The observed ozone threshold is not a clinical decision threshold and should not be used to guide biopsy scheduling. Its relevance is hypothesis-generating: the signal occurred below regulatory short-term limits, but the cutoff was data-driven, single-centre, not externally validated, and associated with any rather than drainage-requiring pneumothorax. Clinical implementation would require prospective evidence of incremental predictive value and net benefit from rescheduling.

Meteorological factors showed no independent association with pneumothorax after accounting for the primary predictors, which is consistent with the only comparable CT-guided lung biopsy study evaluating meteorological conditions [12]. Although studies on primary spontaneous pneumothorax have suggested associations with atmospheric pressure or temperature [34], such effects may be smaller in the procedural setting. In CT-guided lung biopsy, needle trajectory, pleural mechanics, emphysema, and patient positioning may outweigh subtle meteorological variation.

This study has several limitations. Its retrospective single-centre design limits generalisability and may introduce selection bias. A complete comparison of included and excluded patients was not feasible because some exclusions were related to refused or unclear consent, while others reflected predefined criteria for non-comparable procedural conditions or incomplete data. Residual selection bias, therefore, cannot be fully excluded. Exposure assessment was based on a single fixed monitoring station near the hospital and therefore reflected ambient day-of-procedure conditions at the intervention site rather than individual patient-level exposure. We could not account for residence-specific exposure, distance from the hospital, commuting route or mode, time spent indoors before biopsy, ventilation conditions, or attenuation of indoor ozone concentrations. Urban residence served only as a crude surrogate for exposure differences. Although extended sensitivity models jointly adjusted for technical factors, biopsy system, operator identity, and season, residual confounding remains possible, particularly from unmeasured operator technique, subtle parenchymal abnormalities, or patient-level exposure variation. Given the retrospective design, single-station exposure assignment, and threshold-based nature of the ozone finding, the association should not be interpreted as causal. Furthermore, no biomarker, inflammatory marker, lung function, ILD severity score, or direct alveolar mechanics data were available to support the proposed hypothetical mechanism. The drainage-requiring pneumothorax analysis was limited by the small number of events, although Firth penalised regression was used to reduce small-sample bias. Prospective multicentre studies with patient-level exposure assessment are needed to validate these results.

## 5. Conclusions

Ozone exposure showed an exploratory nonlinear association with any pneumothorax after CT-guided lung biopsy, with the strongest threshold signal around 70–80 μg/m^3^. Emphysema was associated with drainage-requiring pneumothorax, and ARDA was consistently protective. Prospective external validation is required before ozone exposure can be considered for procedural risk stratification.

## Figures and Tables

**Table 1:** Cohort characteristics stratified by pneumothorax occurrence. Continuous variables are presented as median [interquartile range] and categorical variables as *n*/*N* (%). *p*-values were calculated using Wilcoxon rank-sum tests for continuous variables and chi-square tests for categorical variables. Pneumothorax was defined as intrapleural gas detected on immediate post-biopsy CT after needle removal. Abbreviations: CT = computed tomography; IQR = interquartile range; y = years; UP = upper lobe; LD = lateral decubitus; IT Afternoon = intervention time later than 12:00 PM; Urban Residence = the patient lives in the city; ARDA = access route begins in dependent area according to our model; COPD = chronic obstructive pulmonary disease; IR = interventional radiologist; G = gauge; SL = skin-to-lesion; PL = pleural-to-lesion; mm = millimeter.

Characteristic	Overall	No Pneumothorax	Pneumothorax	*p*-Value ^2^
	N = 160 ^1^	N = 74 ^1^	N = 86 ^1^	
**Age (y)**	68.5 [60.0, 76.0]	66.5 [55.0, 77.0]	69.0 [63.0, 75.0]	0.2
**Female**	65/160.0 (41%)	34/74.0 (46%)	31/86.0 (36%)	0.3
**Patient Position**				0.089
LD	46/160.0 (29%)	27/74.0 (36%)	19/86.0 (22%)	
Prone	47/160.0 (29%)	17/74.0 (23%)	30/86.0 (35%)	
Supine	67/160.0 (42%)	30/74.0 (41%)	37/86.0 (43%)	
**Lesion Size (mm)**	21.0 [14.0, 30.0]	23.0 [14.0, 30.0]	20.5 [14.0, 30.0]	0.8
**Lesion Location in UP**	75/160.0 (47%)	33/74.0 (45%)	42/86.0 (49%)	0.7
**DPL (mm)**	12.0 [0.0, 26.0]	18.0 [0.0, 26.0]	10.0 [0.0, 23.0]	0.2
**DSL (mm)**	58.5 [45.0, 73.5]	61.0 [46.0, 82.0]	55.5 [44.0, 71.0]	0.2
**Biopsy Angle (degrees)**	66.6 [48.0, 80.5]	68.0 [50.0, 81.0]	64.5 [47.0, 79.0]	0.4
**Needle Size (G)**				<0.001
16	10/160.0 (6%)	2/74.0 (3%)	8/86.0 (9%)	
18	106/160.0 (66%)	41/74.0 (55%)	65/86.0 (76%)	
20	44/160.0 (28%)	31/74.0 (42%)	13/86.0 (15%)	
**Biopsy System**				<0.001
Side-cut	71/160.0 (44%)	20/74.0 (27%)	51/86.0 (59%)	
Full-core	89/160.0 (56%)	54/74.0 (73%)	35/86.0 (41%)	
**Number of Samples (n)**				0.7
1	6/160.0 (4%)	3/74.0 (4%)	3/86.0 (3%)	
2	33/160.0 (21%)	14/74.0 (19%)	19/86.0 (22%)	
3	67/160.0 (42%)	34/74.0 (46%)	33/86.0 (38%)	
4	36/160.0 (23%)	15/74.0 (20%)	21/86.0 (24%)	
5	16/160.0 (10%)	8/74.0 (11%)	8/86.0 (9%)	
6	2/160.0 (1%)	0/74.0 (0%)	2/86.0 (2%)	
**Emphysema**	52/160.0 (33%)	19/74.0 (26%)	33/86.0 (38%)	0.12
**ARDA**	70/160.0 (44%)	46/74.0 (62%)	24/86.0 (28%)	<0.001
**Respiratory Comorbidity**				0.8
None/Unknown	61/160.0 (38%)	28/74.0 (38%)	33/86.0 (38%)	
Asthma	4/160.0 (3%)	1/74.0 (1%)	3/86.0 (3%)	
Smoking	67/160.0 (42%)	33/74.0 (45%)	34/86.0 (40%)	
COPD	28/160.0 (18%)	12/74.0 (16%)	16/86.0 (19%)	
**Intervention After Noon**	58/160.0 (36%)	25/74.0 (34%)	33/86.0 (38%)	0.7
**Season**				0.4
Spring	49/160.0 (31%)	23/74.0 (31%)	26/86.0 (30%)	
Summer	42/160.0 (26%)	15/74.0 (20%)	27/86.0 (31%)	
Fall	32/160.0 (20%)	18/74.0 (24%)	14/86.0 (16%)	
Winter	37/160.0 (23%)	18/74.0 (24%)	19/86.0 (22%)	
**Urban Residence**	54/160.0 (34%)	25/74.0 (34%)	29/86.0 (34%)	>0.9
**Ozone, μg/m^3^**	76.0 [57.4, 95.3]	69.8 [56.8, 87.7]	82.2 [63.4, 100.9]	0.025
**Ozone ≥ 75.8 μg/m^3^**	81/160.0 (51%)	28/74.0 (38%)	53/86.0 (62%)	0.004

^1^ Median [Q1, Q3]; *n*/*N* (%), ^2^ Wilcoxon rank-sum test; Pearson’s chi-square test.

**Table 2:** Primary multivariable logistic regression models for pneumothorax. The primary models included ozone exposure, emphysema, and ARDA. Ozone was analysed both as a linear continuous variable per 10 μg/m^3^ (micrograms per cubic metre) and as a threshold variable using the ROC-derived cutoff of ≥75.8 μg/m^3^. A Firth penalised logistic regression model was additionally fitted for robustness. Odds ratios are reported with 95% confidence intervals. ARDA = access route begins in dependent area according to our model; CI = confidence interval; OR = odds ratio.

Model	Variable	OR 95% CI	*p*-Value
Linear ozone model	Ozone per 10 μg/m^3^	1.09 (0.97–1.23)	0.167
Linear ozone model	Emphysema	1.96 (0.95–4.15)	0.072
Linear ozone model	ARDA	0.23 (0.12–0.45)	0.000
Threshold ozone model	Ozone ≥ 75.8 μg/m^3^	2.76 (1.39–5.61)	0.004
Threshold ozone model	Emphysema	2.16 (1.03–4.68)	0.047
Threshold ozone model	ARDA	0.23 (0.11–0.45)	0.000
Firth threshold model	Ozone ≥ 75.8 μg/m^3^	2.68 (1.37–5.39)	0.004
Firth threshold model	Emphysema	2.1 (1.01–4.50)	0.047
Firth threshold model	ARDA	0.24 (0.12–0.46)	0.000

**Table 3:** Robustness analyses for predictors of pneumothorax. The base threshold model included ozone ≥ 75.8 μg/m^3^, emphysema, and ARDA. Sensitivity models additionally adjusted for selected procedural or contextual covariates, including needle size, biopsy system, operator identity, and season. Values are odds ratios with 95% confidence intervals and *p*-values. ARDA = access route begins in dependent area according to our model; CI = confidence interval; OR = odds ratio.

Model	Ozone ≥ 75.8 μg/m^3^	Emphysema	ARDA
Base model	2.76 (1.39–5.61); *p* = 0.004	2.16 (1.03–4.68); *p* = 0.046	0.23 (0.11–0.45); *p* ≤ 0.001
Needle size adjusted	2.87 (1.40–6.07); *p* = 0.005	2.18 (0.99–4.94); *p* = 0.056	0.23 (0.11–0.48); *p* ≤ 0.001
Biopsy system adjusted	2.93 (1.43–6.18); *p* = 0.004	2.19 (1.01–4.93); *p* = 0.051	0.27 (0.13–0.56); *p* ≤ 0.001
Operator adjusted	2.53 (1.11–5.94); *p* = 0.029	1.66 (0.71–3.93); *p* = 0.243	0.22 (0.10–0.49); *p* ≤ 0.001
Season adjusted	2.96 (1.32–6.87); *p* = 0.009	2.11 (0.99–4.63); *p* = 0.056	0.23 (0.11–0.46); *p* ≤ 0.001

**Table 4:** Firth logistic regression for drainage-requiring pneumothorax. Drainage-requiring pneumothorax was defined as pneumothorax requiring placement of a pleural drainage catheter after CT-guided lung biopsy. Because of the limited number of drainage events, Firth penalised logistic regression was used. Odds ratios are reported with 95% confidence intervals. ARDA = access route begins in dependent area according to our model; CI = confidence interval; OR = odds ratio.

Variable	OR (95% CI)	*p*-Value
Ozone ≥ 75.8 μg/m^3^	1.63 (0.53–5.41)	0.399
Emphysema	3.62 (1.19–11.87)	0.023
ARDA	0.85 (0.26–2.63)	0.778

## Data Availability

The data presented in this study are available from the corresponding author upon reasonable request. The data are not publicly available due to patient privacy, institutional data protection regulations, and restrictions imposed by the ethics approval.

## Supplementary Materials

The following supporting information can be downloaded at: https://www.mdpi.com/article/10.3390/tomography12070098/s1, Supplementary Table S1: Full ozone-threshold sensitivity analysis; Supplementary Table S2: Operator distribution and pneumothorax rates; Supplementary Table S3: Extended confounding sensitivity models. Supplementary Figure S1: Hypothetical conceptual pathway linking ambient ozone exposure to increased lung vulnerability during CT-guided lung biopsy. Supplementary Figure S2: Illustrative cases with different maximum hourly mean ambient ozone exposure.

## Abbreviations

The following abbreviations are used in this manuscript:

ARDA	Access route through dependent lung area
CI	Confidence interval
CO	Carbon monoxide
COPD	Chronic obstructive pulmonary disease
CT	Computed tomography
CTLB	Computed tomography-guided lung biopsy
DPL	Distance from pleura to lesion
DSL	Distance from skin to lesion
EC	Elemental carbon
G	Gauge
IQR	Interquartile range
LD	Lateral decubitus
NO_2_	Nitrogen dioxide
NO_x_	Nitrogen oxides
OR	Odds ratio
PM_10_	Particulate matter with an aerodynamic diameter ≤ 10 μm
PM_2.5_	Particulate matter with an aerodynamic diameter ≤ 2.5 μm
PPL	Pleural pressure
ROC	Receiver operating characteristic
TLA	Three-letter acronym
UP	Upper lobe
