# Supplementary material for: Ambient Ozone Exposure and Pneumothorax Risk After CT-Guided Lung Biopsy
Source: Tomography. 2026 Jul 1;12(7):98. doi: 10.3390/tomography12070098 (PMC13416874; doi:10.3390/tomography12070098)
Supplement: Supplementary file 1 [file tomography-12-00098-s001.zip › tomography-4362697-supplementary.pdf]

Ozone threshold, μg/m³	n below	n above	Pneumothorax below, n	Pneumothorax above, n	Risk below, %	Risk above, %	Adjusted OR (95% CI)	*p* value
60	43	117	21	65	48.8	55.6	1.26 (0.59–2.69)	0.546
65	50	110	22	64	44	58.2	2.02 (0.98–4.27)	0.06
70	62	98	25	61	40.3	62.2	2.60 (1.29–5.33)	0.008
75	71	89	28	58	39.4	65.2	2.78 (1.40–5.62)	0.004
75.8	79	81	33	53	41.8	65.4	2.76 (1.39–5.61)	0.004
80	86	74	39	47	45.3	63.5	2.02 (1.02–4.04)	0.044
85	98	62	47	39	48	62.9	1.94 (0.97–3.98)	0.064
90	112	48	55	31	49.1	64.6	1.97 (0.94–4.27)	0.078
95	120	40	58	28	48.3	70	2.50 (1.12–5.86)	0.028
100	130	30	64	22	49.2	73.3	2.42 (0.98–6.48)	0.065
110	146	14	76	10	52.1	71.4	1.83 (0.53–7.47)	0.361
120	153	7	80	6	52.3	85.7	2.48 (0.38–48.47)	0.416

Supplementary Table S1. Full ozone-threshold sensitivity analysis. Threshold models were fitted using alternative ozone cut-offs from 60 to 120 μg/m³. Each model was adjusted for emphysema and ARDA. Event rates below and above each threshold are shown together with adjusted odds ratios, 95 % confidence intervals, and p values. ARDA = access route begins in dependent area according to our model; CI, confidence interval; OR, odds ratio.

operator	n	pneumothorax (n)	pneumothorax (%)	high_ozone (n)	high_ozone (%)
A	11	5	45.5	4	36.4
B	33	26	78.8	28	84.8
C	11	9	81.8	6	54.5
D	10	6	60	5	50
E	4	2	50	0	0
F	42	8	19	16	38.1
G	24	13	54.2	15	62.5
H	25	17	68	7	28

Supplementary Table S2. Operator distribution and pneumothorax rates. The number of procedures, pneumothorax events, and high-ozone procedures are shown for each operator. Operators are anonymised. High ozone was defined as ozone ≥75.8 μg/m³.

Model	Ozone	Emphysema	ARDA
Fully adjusted: technique + operator + season	2.93 (1.12–7.97); *p*=0.030	1.73 (0.72–4.25); *p*=0.226	0.20 (0.08–0.47); *p*<0.001
Fully adjusted: technique + operator + season + anatomy	2.91 (1.11–7.92); *p*=0.032	1.73 (0.72–4.28); *p*=0.225	0.20 (0.08–0.48); *p*<0.001
Sparse operator model	2.57 (1.05–6.49); *p*=0.039	1.64 (0.72–3.79); *p*=0.237	0.25 (0.11–0.54); *p*<0.001

Supplementary Table S3. Extended confounding sensitivity models.
Extended sensitivity models evaluated whether the association between ozone ≥75.8 μg/m³ and pneumothorax persisted after simultaneous adjustment for technical, operator-related, seasonal, and anatomic covariates. The fully adjusted model included ozone ≥75.8 μg/m³, emphysema, ARDA, needle size, biopsy system, operator identity, and season. The fully adjusted anatomic model additionally included lesion size and pleura-to-lesion distance. The sparse operator model replaced individual operator identity with a binary indicator of above-median operator-specific pneumothorax rate. Values are odds ratios with 95% confidence intervals and p values. ARDA = access route begins in dependent area according to our model; CI, confidence interval; OR, odds ratio.

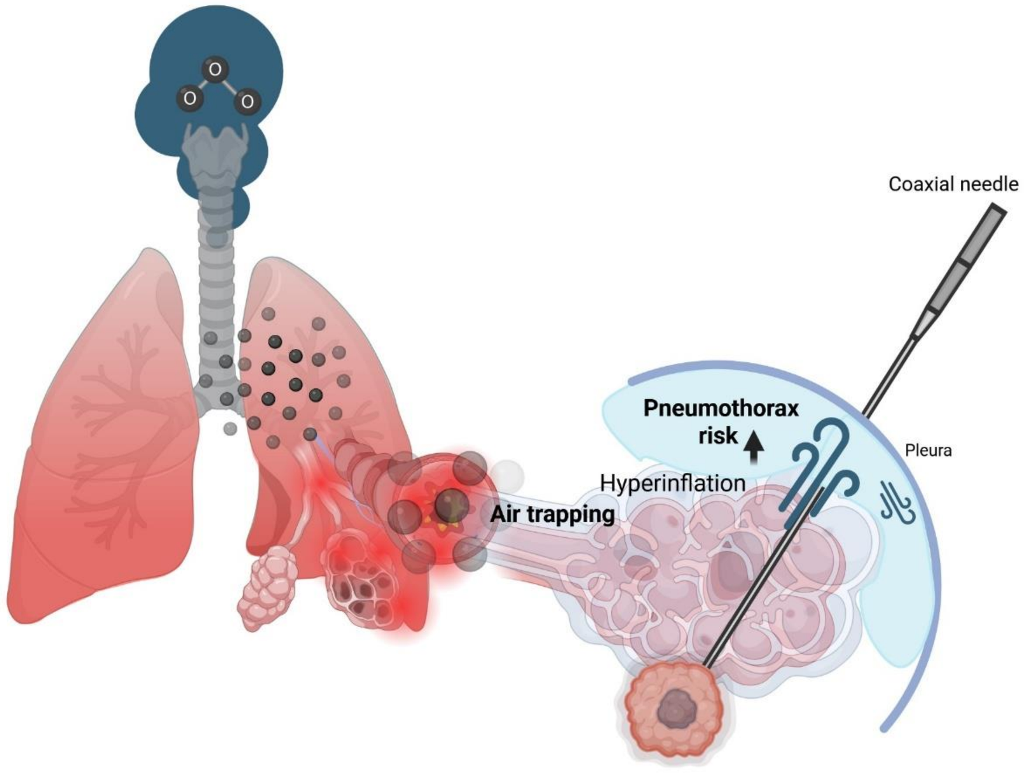

Supplementary Figure S1. Hypothetical conceptual pathway linking ambient ozone exposure to increased lung vulnerability during CT-guided lung biopsy. Ozone may contribute to oxidative stress, epithelial injury, airway inflammation, bronchiolar narrowing, air trapping, and alveolar overdistension, which could theoretically increase susceptibility to alveolar rupture during coaxial needle biopsy. This mechanism was not directly tested in the present retrospective observational study and requires validation in dedicated mechanistic studies.

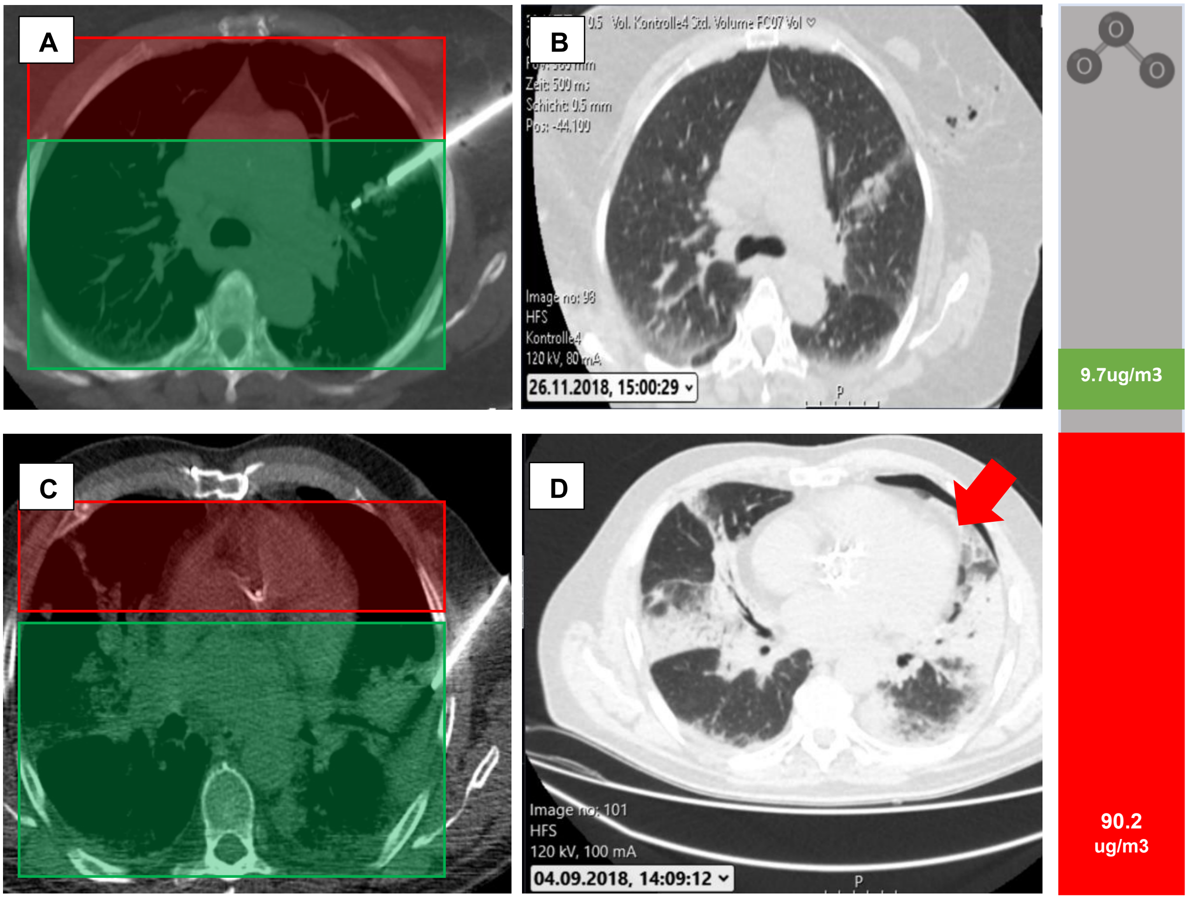

Supplementary Figure S2. Illustrative cases with different maximum hourly mean ambient ozone exposure. Representative CT-guided lung biopsy cases with different ozone exposure levels on the day of intervention. **A–B**, biopsy performed with the access route within the dependent lung region and without subsequent pneumothorax at a maximum hourly mean ozone concentration of 9.7 μg/m³. **C–D**, biopsy performed under similar positioning and technique with subsequent left-sided pneumothorax at a maximum hourly mean ozone concentration of 90.2 μg/m³. These cases are illustrative only and do not establish causality.
